# Effect of TMAO on the incidence and prognosis of cerebral infarction: a systematic review and meta-analysis

**DOI:** 10.3389/fneur.2023.1287928

**Published:** 2024-01-08

**Authors:** Lin Wang, Yinan Nan, Wenhao Zhu, Shaoqing Wang

**Affiliations:** ^1^Traditional Chinese Medicine Department, Beijing Tiantan Hospital, Beijing, China; ^2^International Department, China-Japan Friendship Hospital, Beijing, China; ^3^Department of Encephalopathy, Zibo Hospital of Traditional Chinese Medicine, Zibo, China

**Keywords:** cerebral infarction, TMAO, prognosis, morbidity, meta-analysis, systematic evaluation

## Abstract

**Objective:**

This study aimed to evaluate the effect of trimethylamine oxide (TMAO) on the incidence and prognosis of cerebral infarction.

**Methods:**

We searched PubMed, Embase, and Cochrane databases for all clinical studies on the association of TMAO with cerebral infarction incidence and prognosis from inception to April 2023. A systematic review and meta-analysis were conducted using the meta-analysis of observational studies in epidemiology (MOOSE) declaration list. The Newcastle–Ottawa Scale (NOS) was used to assess the quality of the study. This study protocol was registered on the PROSPERO database with the ID: CRD42023459661. The extracted data included the OR value of the effect of TMAO on the incidence and prognosis of cerebral infarction, the HR value between TMAO and underlying diseases, the RR value, 95% confidence intervals, and the AUC value of TMAO in the prediction model of cerebral infarction.

**Results:**

Fifteen studies including 40,061 patients were included. All the patients were from China or Germany. The TMAO level was significantly correlated with the Modified Rankin Score (mRS) 3 months after the onset of cerebral infarction (OR, 1.581; 95% CI, 1.259–1.987; *p* < 0.01). The TMAO level was significantly correlated with the rate of first-time incidence and recurrence of cerebral infarction (OR, 1.208; 95% CI, 1.085–1.344; *p* < 0.01 and HR, 1.167; 95% CI, 1.076–1.265; *p* < 0.01, respectively). The TMAO level was also highly correlated with disease severity at onset (National Institutes of Health Stroke Scale, NIHSS >5) (OR, 5.194; 95% CI, 1.206–22.363; *p <* 0.05), but had no significant correlation with mortality after cerebral infarction (*p* > 0.05). Correlation analysis of TMAO with underlying diseases in the population indicated that TMAO had a significant correlation with histories of hypertension, diabetes mellitus, coronary artery disease, and cerebral infarction (*p* < 0.05), but not with hyperlipidemia (*p* > 0.05). Six risk prediction models of TMAO for cerebral infarction reported in four studies were systematically evaluated; five of them had good predictive value (AUC ≥ 0.7).

**Conclusion:**

TMAO is an independent risk factor affecting the onset, prognosis, and severity of cerebral infarction.

## 1 Introduction

Globally, stroke was the second leading cause of death and the third leading cause of death and disability in 2019. From 1990 to 2019, the absolute number of incident strokes, prevalent strokes, and deaths from stroke increased by 70, 85, and 43%, respectively (1). While controlling the primary disease, the need to implement new and effective prevention and control measures for stroke occurrence, development, and recurrence is urgent. Some studies have shown that intestinal flora can not only protect the body by activating the human immune system but also attack the body by altering the structure of the flora resulting in tumors, endocrine system disorders, and cardiovascular and cerebrovascular events (2). In recent years, the correlation between intestinal flora metabolism and the incidence and prognosis of cerebral infarction has attracted increasing attention (3). Trimethylamine oxide (TMAO) is a small molecule produced from dietary choline, lecithin, and l-carnitine, which are mostly found in ingredients such as eggs, fish, and red meat (4). The levels of TMAO in the human body are regulated by a variety of factors, including diet, host genotype, and intestinal flora composition (5). Recent studies have shown that large artery atherosclerotic cerebral infarction and coronary heart disease share common risk factors and pathophysiological bases and that elevated TMAO concentrations are associated with atherosclerosis and cardiovascular disease (6, 7).

Some studies have shown that the intestinal flora compositions and TMAO levels of patients with cerebral infarction are significantly different from those of healthy people and that TMAO plays an important role in the process of atherosclerosis and vascular embolic events (8). An increasing number of studies have focused on the correlation between TMAO and cerebral infarction including its influence on the onset, prognosis, and severity of cerebral infarction; the results of this research have not reached a consensus. This meta-analysis and systematic evaluation of the correlation between TMAO and the incidence of cerebral infarction aimed to clarify the role of TMAO in influencing the development and prognosis of cerebral infarction.

## 2 Materials and methods

### 2.1 Literature search

This study was rigorously designed and executed in strict accordance with the guidelines of the preferred reporting items for the meta-analysis of observational studies in epidemiology (MOOSE) declaration list. Comprehensive searches of PubMed, Embase, and Cochrane databases from inception to April 2023 were conducted to formulate subject headings and free terms, with the following search strategy: “TMAO OR Trimethyloxamine OR trimethylamine-n-oxide OR trimethylamine oxide” AND “Ischemic Stroke OR cerebral infarction OR brain infarction OR acute cerebral infarction OR infarction OR cerebral infarct OR cerebral ischemia.” The citation lists of the retrieved articles were manually screened to ensure the sensitivity of the search strategy. Only English literature was included.

### 2.2 Inclusion and exclusion criteria

Inclusion criteria were as follows: clinical studies, including randomized controlled trials (RCTs), cohort studies, and case–control studies; study population of patients with cerebral infarction definitively diagnosed by computed tomography (CT) or magnetic resonance imaging (MRI) and not combined with other serious organic diseases and complications; primary outcome metrics, including the odds ratio (OR) of the Modified Rankin Score (mRS) at 3 months after cerebral infarction predicted by TMAO, OR of cerebral infarction onset predicted by TMAO, hazard ratio (HR) of recurrence predicted by TMAO, OR of the correlation between TMAO and severity of neurological deficits at onset of cerebral infarction (National Institutes of Health Stroke Scale [NIHSS] > 5), OR of the effect of TMAO on mortality after cerebral infarction, relative risk (RR) of the correlation between TMAO and patients with cerebral infarction with underlying disease correlation, and area under the receiver operating characteristic curve (AUC) of the risk prediction model of TMAO on cerebral infarction.

The exclusion criteria were as follows: duplicate publications; animal experiments; review papers; conference papers; literature for which the full text was not available; and literature for which the data were incomplete, and data merging was not possible.

### 2.3 Literature screening, data organization, and data extraction

Literature screening was conducted as follows: Two researchers read the titles and abstracts of the studies and judged whether to include the studies by reading the full text according to the inclusion and exclusion criteria. This process was conducted independently. In the event of disagreement during the selection process, a third person made the final decision.

Data organization was conducted as follows: The literature organization mainly included literature type, basic characteristics of study subjects, specific levels of TMAO, OR values, HR values, RR values, AUC values of the logistic regression model, and 95% confidence intervals (Cis).

Data extraction and quality evaluation were conducted as follows: All article data were extracted and evaluated by two independent researchers. For each study, data were extracted, including the first author, year of publication, sample size, control group, serum TMAO level, OR, HR, RR, AUC of the logistic regression model, and 95% CI. After completion, the study was checked to prevent omissions and errors.

Literature quality evaluation was conducted as follows: Two researchers simultaneously applied the Newcastle–Ottawa Scale (NOS) to evaluate the quality of the literature, and divergent literature was referred to a third person to reach a decision.

### 2.4 Quality assessment

Studies were assessed using the NOS, and a score greater than 6 was considered high-quality literature (9).

### 2.5 Statistical methods

For quantitative analysis, we used the “metan” package based on the STATA statistical software for meta-analyses. The meta-analysis used ORs or HRs and 95% CIs to aggregate effect size. The tau square (τ^2^) test and value of p were used to qualitatively analyze the statistical heterogeneity between the studies. A larger τ^2^ and a smaller *p*-value indicated a greater possibility of heterogeneity. In addition, *I*^2^ is a parameter used for the quantitative analysis of heterogeneity between the results of each study and is distributed from 0 to 100%. When *I*^2^ > 50%, large heterogeneity was indicated. When *p* > 0.1 and *I*^2^ ≤ 50%, the fixed-effects model was applied. When *p* ≤ 0.1 and *I*^2^ > 50%, the random-effects model was applied. The sensitivity of our findings was assessed by repeating each meta-analysis after excluding high-risk studies. A funnel plot (>10 studies) was used to assess the risk of publication bias under specific circumstances when feasible, and Egger’s test suggested publication bias when *p* < 0.05. Different study types were counted separately.

## 3 Results

### 3.1 Results of literature screening

**Figure 1:**
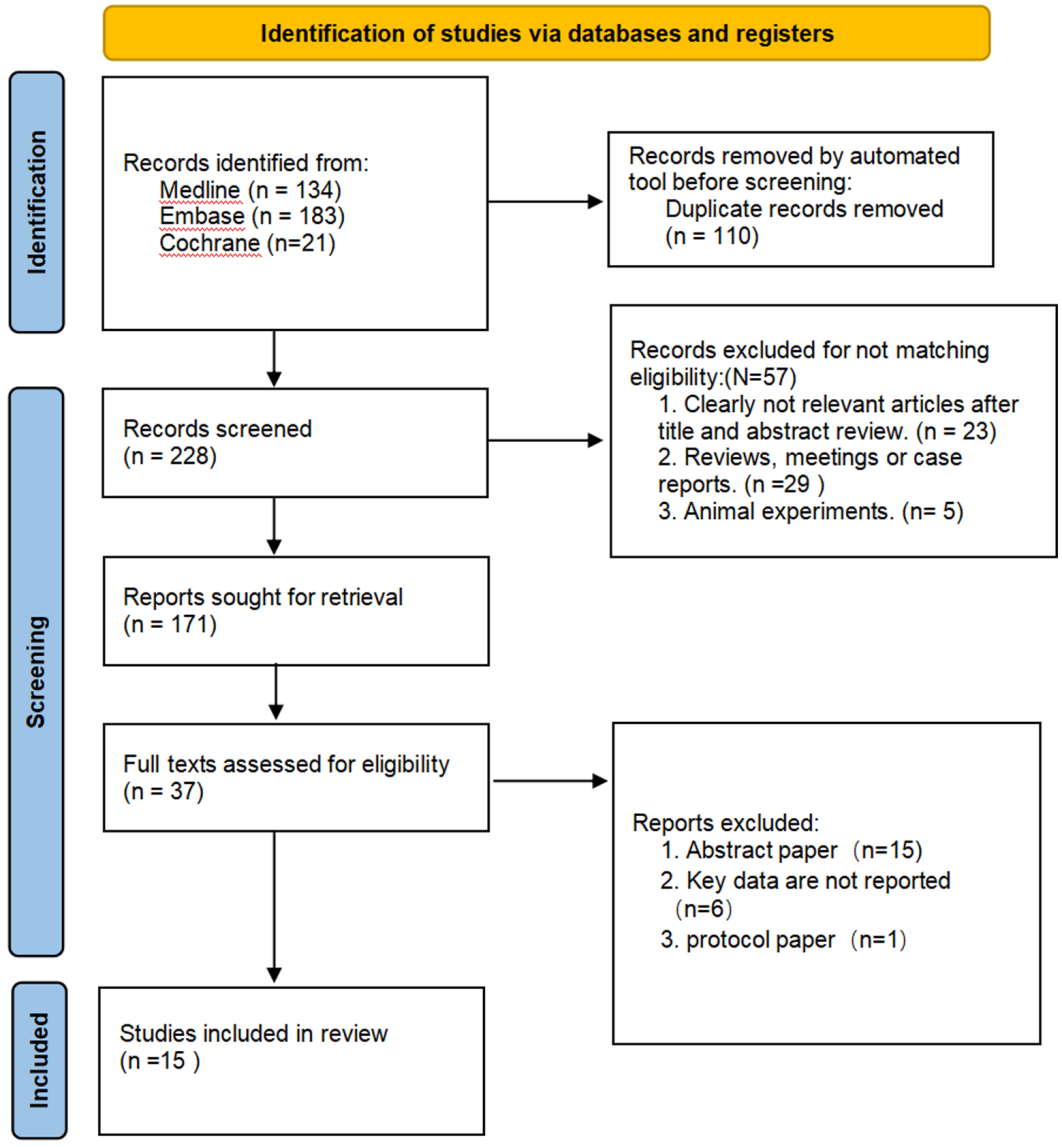
Flow diagram showing the search and screening process.

In this study, we screened the literature according to the MOOSE process and initially retrieved 338 articles from Medline (*n* = 134), Embase (*n* = 183), and Cochrane (*n* = 21). Bibliographic titles were imported into the bibliographic management software Endnote to remove 110 duplicate records and 228 articles proceeded to the next stage. After reading the titles and abstracts, 57 articles were excluded from the analysis. After reading the full texts of the remaining literature to screen according to the inclusion criteria, 134 records were eliminated. Additionally, 15 articles were reviews or meetings, the OR/HR values of 6 studies were not reported, and 1 was an animal experiment (Figure 1 and Table 1), which finally included 15 pieces of literature.

**Table 1:** Baseline information of literature included.

References	Country	Research type	Endpoint criteria	*N*
Tan et al. (10)	China	Cohort study	OR value of poor mRS Score in 3 months	204
Zhang et al. (11)	China	Cohort study	OR value of poor mRS Score in 3 months; OR value for cerebral infarction mortality	351
Zhai et al. (12)	China	Cohort study	OR value of poor mRS Score in 3 months; OR value for cerebral infarction mortality; RR value for morbidity of hypertension, diabetes, CHD, cerebral infarction, hyperlipemia	225
Rexidamu et al. (13)	China	Cohort study	OR value of poor mRS Score in 3 months; OR value for NIHSS>5	255
Chen et al. (14)	China	Case–control study	OR value of poor mRS Score in 3 months; OR value for morbidity of cerebral infarction; RR value for morbidity of hypertension, diabetes	291
Schneider et al. (15)	Germany	Case–control study	OR value of poor mRS Score in 3 months	296
Xu et al. (16)	China	Registry-based study	HR value of cerebral infarction recurrence; RR value for morbidity of hypertension, diabetes, CHD, cerebral infarction, hyperlipemia	10,027
Sun et al. (17)	China	Case–control study	OR value of morbidity of cerebral infarction	1906
Liu et al. (18)	China	Nested case–control studies	OR value of morbidity of cerebral infarction	642
Chen et al. (19)	China	Case–control study	OR value of morbidity of cerebral infarction	610
Xue et al. (20)	China	Registry-based study	HR value of cerebral infarction recurrence; RR value for morbidity of hypertension, CHD, cerebral infarction	9,793
Heyse et al. (21)	Germany	Case–control study	OR value of morbidity of cerebral infarction	82
Xu et al. (22)	China	Registry-based study	HR value of cerebral infarction recurrence	10,756
Xu et al. (23)	China	Registry-based study	HR value of cerebral infarction recurrence; RR value for morbidity of hypertension, diabetes, CHD, cerebral infarction, hyperlipemia	4,186
Wu et al. (24)	China	Case–control study	OR value for NIHSS>5	427

### 3.2 Basic characteristics of included literature and literature quality evaluation

Of the 15 studies, 7 were case–control studies, 4 were cohort studies, and 4 were enrollment studies. The quality of the literature was evaluated using the NOS. All studies analyzed were of high quality (NOS ≥ 6) (Table 2).

**Table 2:** The Newcastle–Ottawa Quality Assessment Scale for included controlled studies.

References	Selection of the study groups	Comparability of the groups	Outcome	Total score
Tan et al. (10)	⭐⭐⭐⭐	⭐	⭐⭐⭐	8
Zhang et al. (11)	⭐⭐⭐	⭐	⭐⭐⭐	7
Zhai et al. (12)	⭐⭐⭐⭐	⭐	⭐⭐⭐	8
Rexidamu et al. (13)	⭐⭐⭐	⭐⭐	⭐⭐⭐	8
Chen et al. (14)	⭐⭐⭐⭐	⭐⭐	⭐⭐⭐	9
Schneider et al. (15)	⭐⭐⭐⭐	⭐⭐	⭐⭐⭐	9
Xu et al. (16)	⭐⭐⭐⭐	⭐⭐	⭐⭐⭐	9
Sun et al. (17)	⭐⭐⭐⭐	⭐⭐	⭐⭐⭐	9
Liu et al. (18)	⭐⭐⭐	⭐	⭐⭐⭐	7
Chen et al. (19)	⭐⭐⭐	⭐	⭐⭐⭐	7
Xue et al. (20)	⭐⭐⭐⭐	⭐	⭐⭐⭐	8
Heyse et al. (21)	⭐⭐⭐	⭐⭐	⭐⭐⭐	8
Xu et al. (22)	⭐⭐⭐⭐	⭐⭐	⭐⭐⭐	9
Xu et al. (23)	⭐⭐⭐⭐	⭐⭐	⭐⭐⭐	9
Wu et al. (24)	⭐⭐⭐⭐	⭐⭐	⭐⭐⭐	9

### 3.3 Meta-analysis results

#### 3.3.1 Relationship between TMAO and 3-month prognosis of cerebral infarction

**Figure 2:**
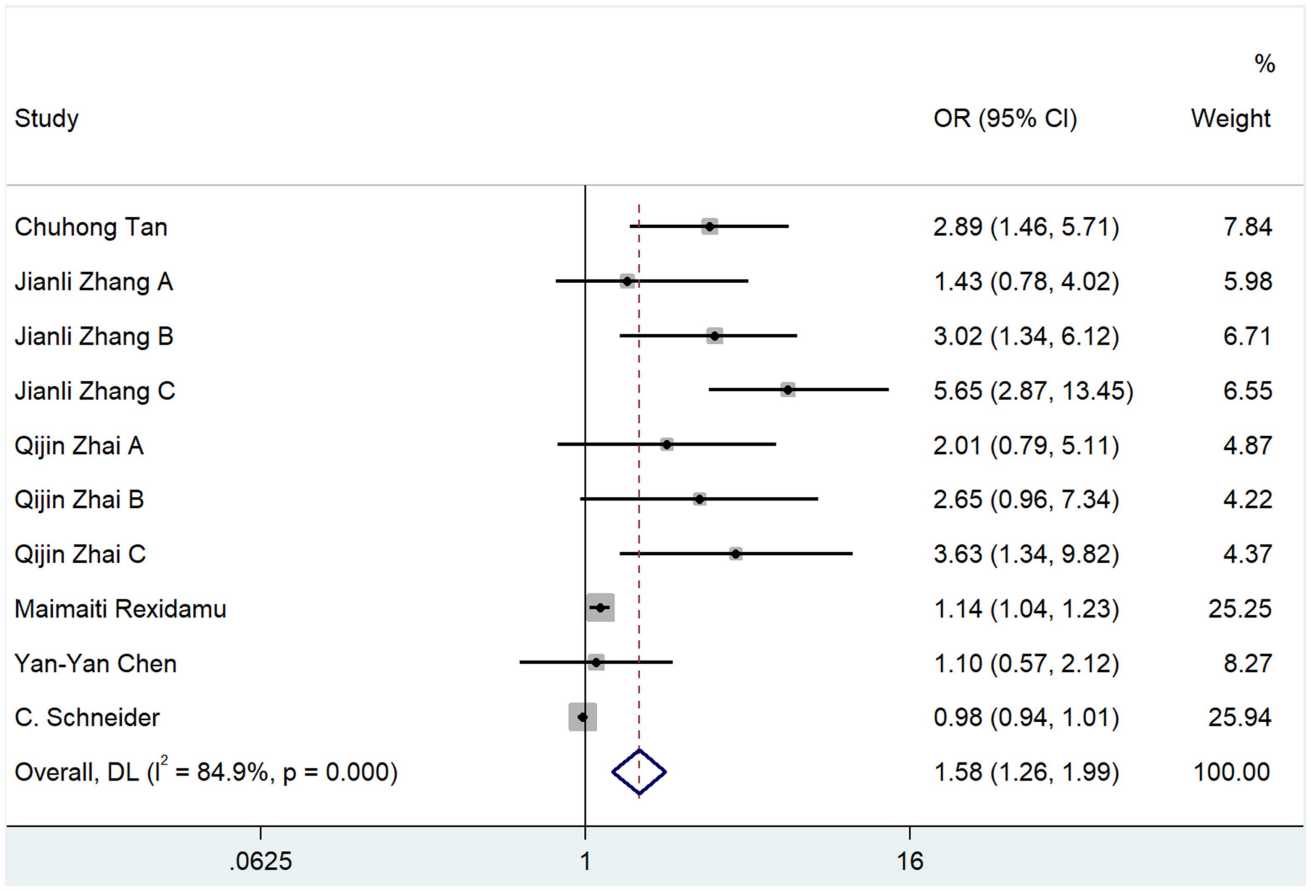
Relationship between TMAO and mRS score at 3 months after cerebral infarction.

Of the 15 studies included in this review, 6 used mRS scores 3 months after the onset of cerebral infarction as the outcome index, and 1,533 subjects were included. The heterogeneity test resulted in *p* = 0.000 and *I*^2^ = 84.9%, and a random-effects model was used for the analysis. The results indicated that TMAO was a risk factor for poor mRS scores at 3 months after cerebral infarction (OR, 1.581; 95% CI, 1.259–1.987; Z = 3.936; *p* = 0.000) (Figure 2).

#### 3.3.2 Correlation analysis between TMAO and the development of cerebral infarction

Of the 15 studies included in this review, 9 studies used cerebral infarction onset or recurrence as an outcome indicator, among which 4 studies reported HR values and 5 studies reported OR values, involving 38,528 cases.

**Figure 3:**
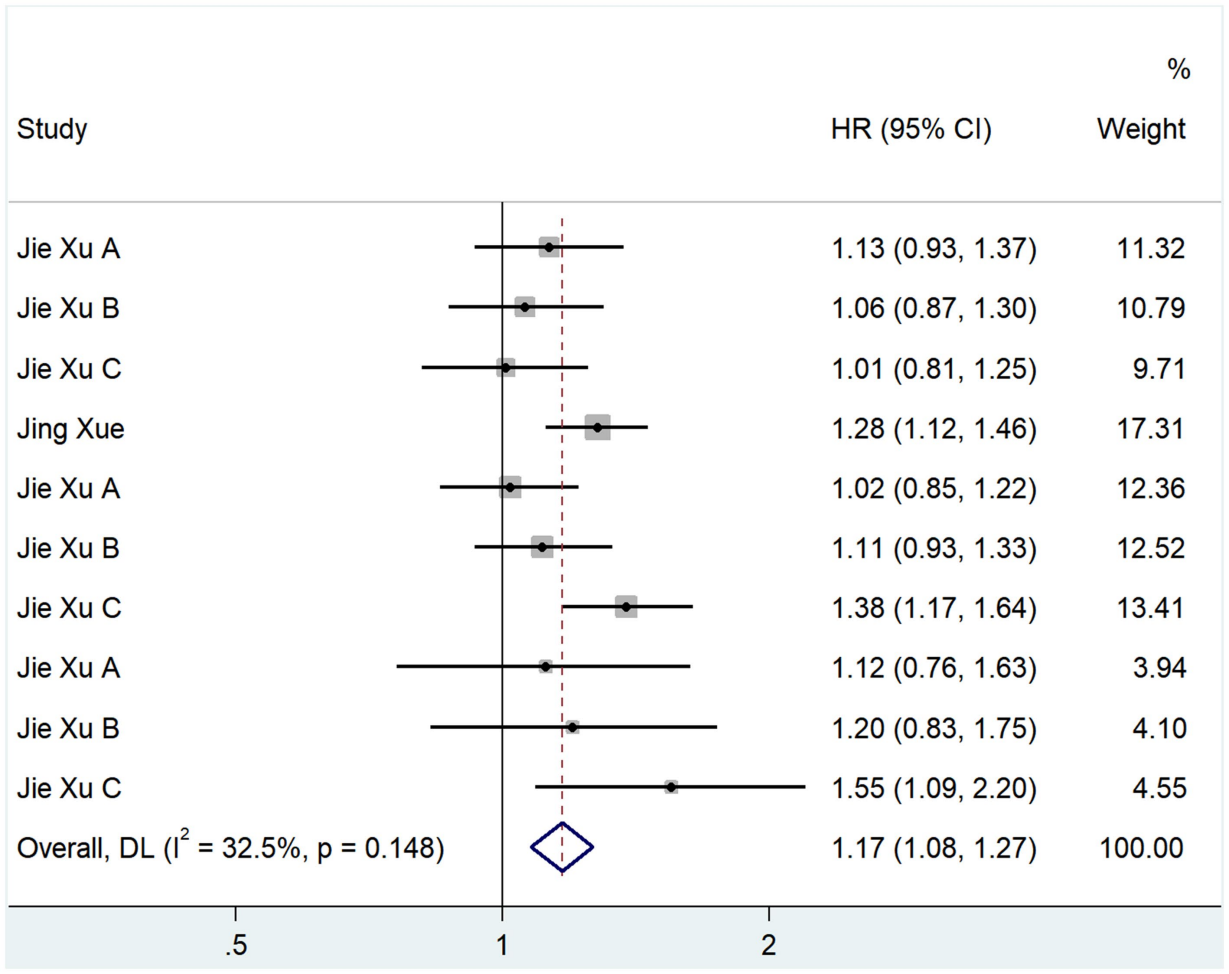
Relationship between TMAO and recurrence of cerebral infarction.

We evaluated the relationship between TMAO and cerebral infarction recurrence using HR values. In total, 4 studies reported HR values; the heterogeneity test showed that *p* = 0.148 and *I*^2^ = 32.5% (Figure 3). A fixed-effects model analysis was used. The results showed that TMAO was a risk factor for cerebral infarction recurrence (HR, 1.167; 95% CI, 1.076–1.265; *Z* = 3.747; *p* = 0.000).

**Figure 4:**
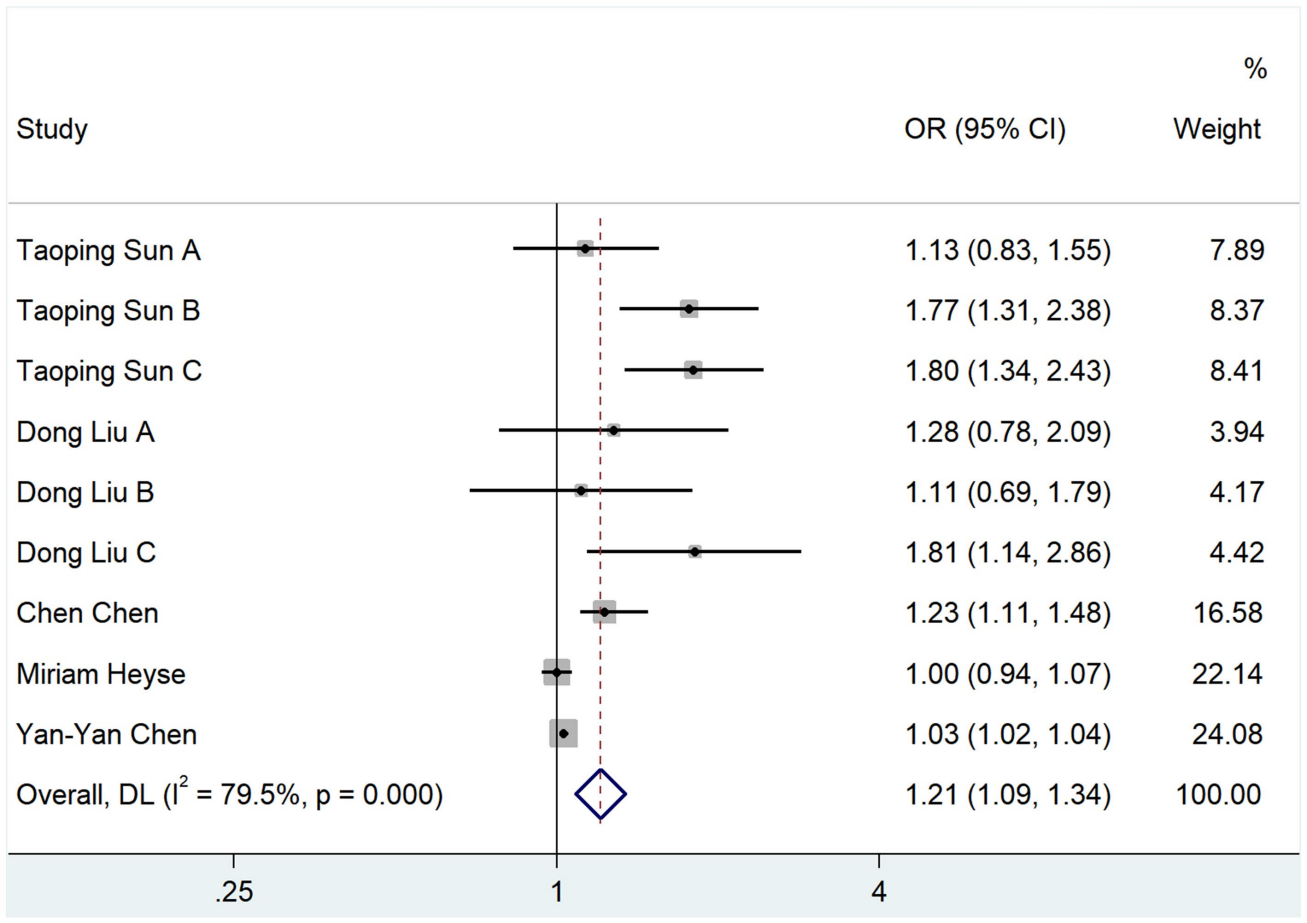
Relationship between TMAO and morbidity of cerebral infarction.

The relationship between TMAO and the onset of cerebral infarction was analyzed using the OR scale. In total, 5 studies reported OR values; the heterogeneity test showed that *p* = 0.000 and *I*^2^ = 79.5%. The results of the random-effects model analysis showed that TMAO was a risk factor for the development of cerebral infarction (OR, 1.208; 95% CI, 1.085–1.344; *Z* = 3.458, *p* = 0.001) (Figure 4).

#### 3.3.3 Effect of TMAO on the severity of cerebral infarction development

**Figure 5:**
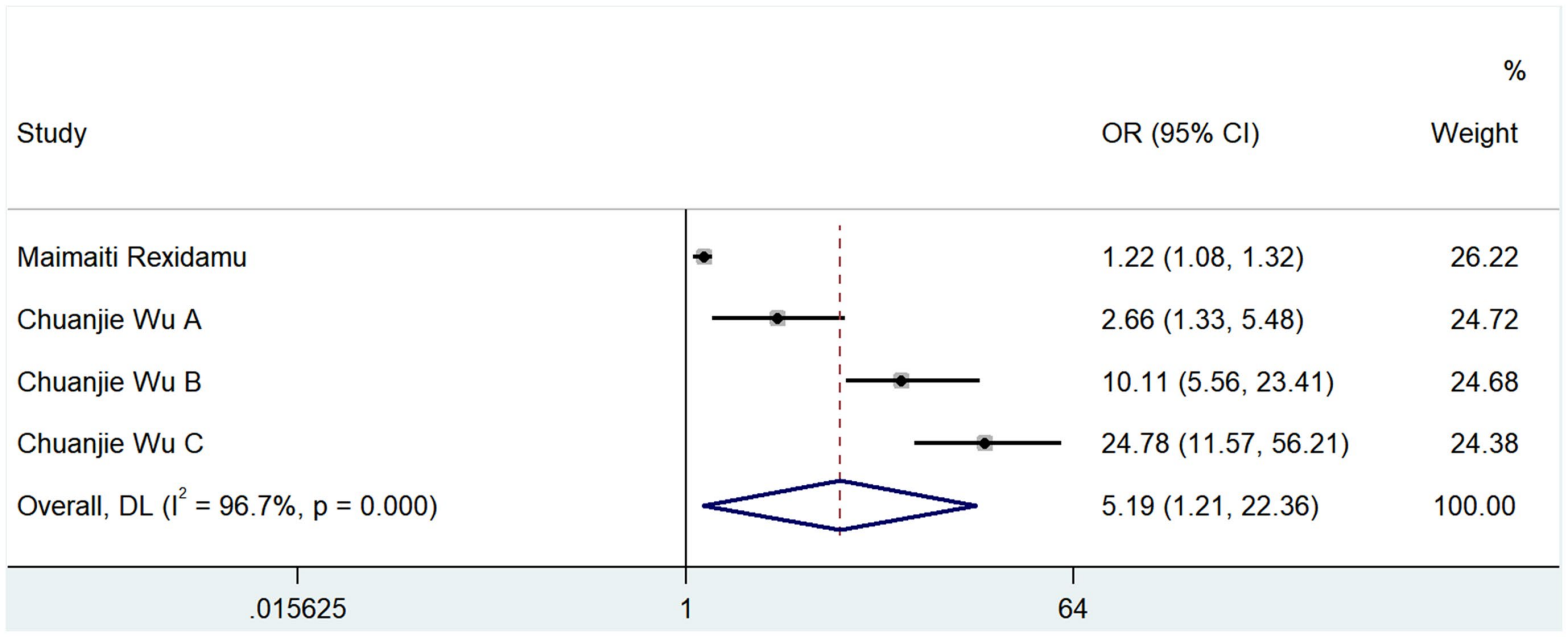
Effect of TMAO on the severity of cerebral infarction.

The severity of cerebral infarction was categorized as mild (NIHSS ≤ 5) or moderately severe (NIHSS > 5) based on the NIHSS score. Two studies reported correlations between TMAO and the severity of neurological deficits at the onset of cerebral infarction; the results of the heterogeneity test showed that *p* = 0.000 and *I*^2^ = 96.7% (Figure 5). The results of the random-effects model analysis (OR, 5.194; 95% CI, 1.206–22.363; *Z* = 2.212, *p* = 0.027) showed that TMAO was a risk factor for severe neurological deficits in cerebral infarction.

#### 3.3.4 Effect of TMAO on mortality from cerebral infarction

**Figure 6:**
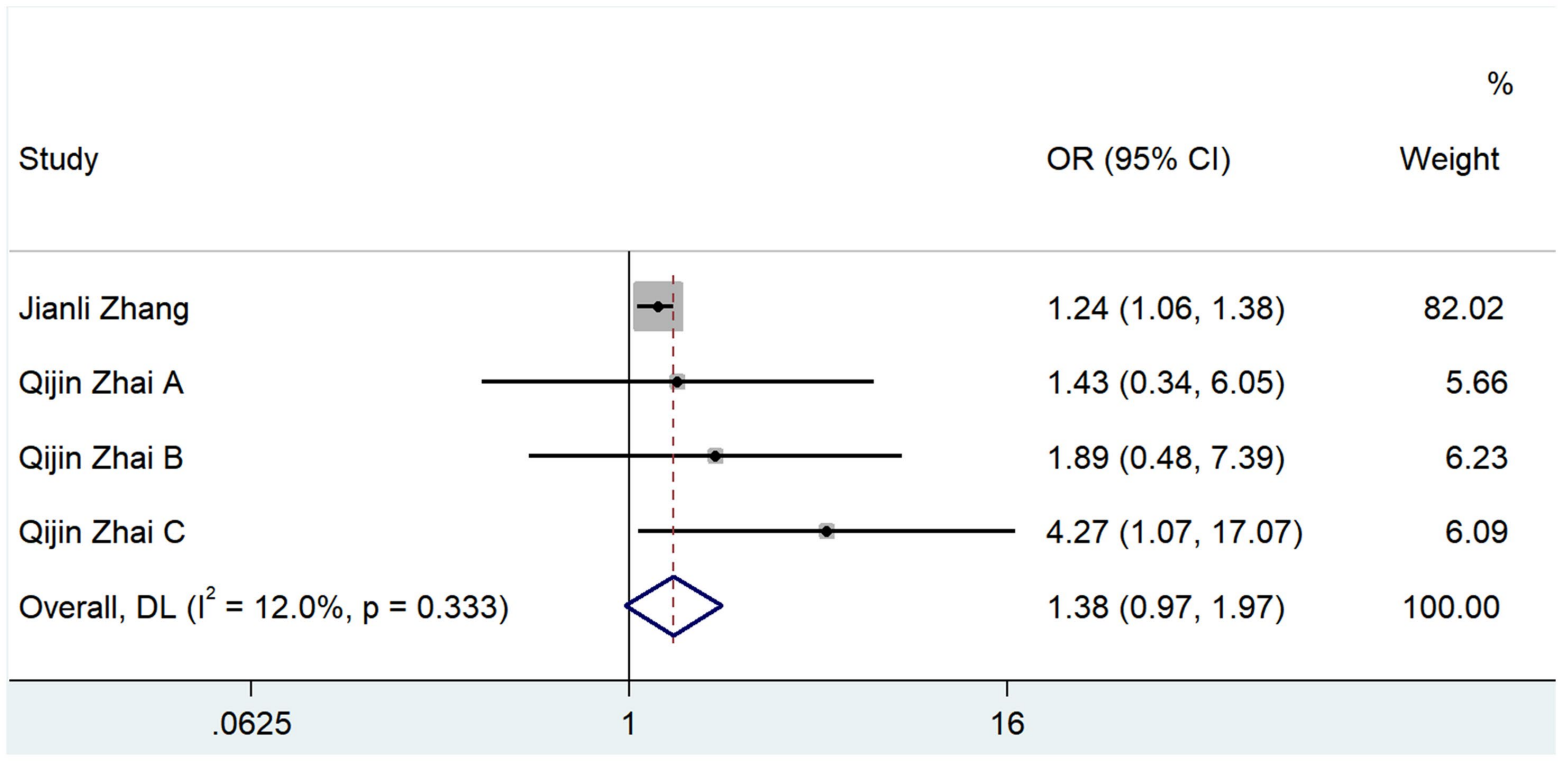
Effect of TMAO on mortality of cerebral infarction.

Two studies reported the ORs of the effects of TMAO and mortality after cerebral infarction. The results of the heterogeneity test showed that *p* = 0.333 and *I*^2^ = 12%, and the results of the fixed-effects model (OR, 1.384; 95% CI, 0.972–1.970; *Z* = 1.081; *p* = 0.072) showed that TMAO was not significantly correlated with the mortality rate of cerebral infarction (Figure 6).

### 3.4 Correlation analysis between TMAO and underlying diseases

#### 3.4.1 Hypertension

**Figure 7:**
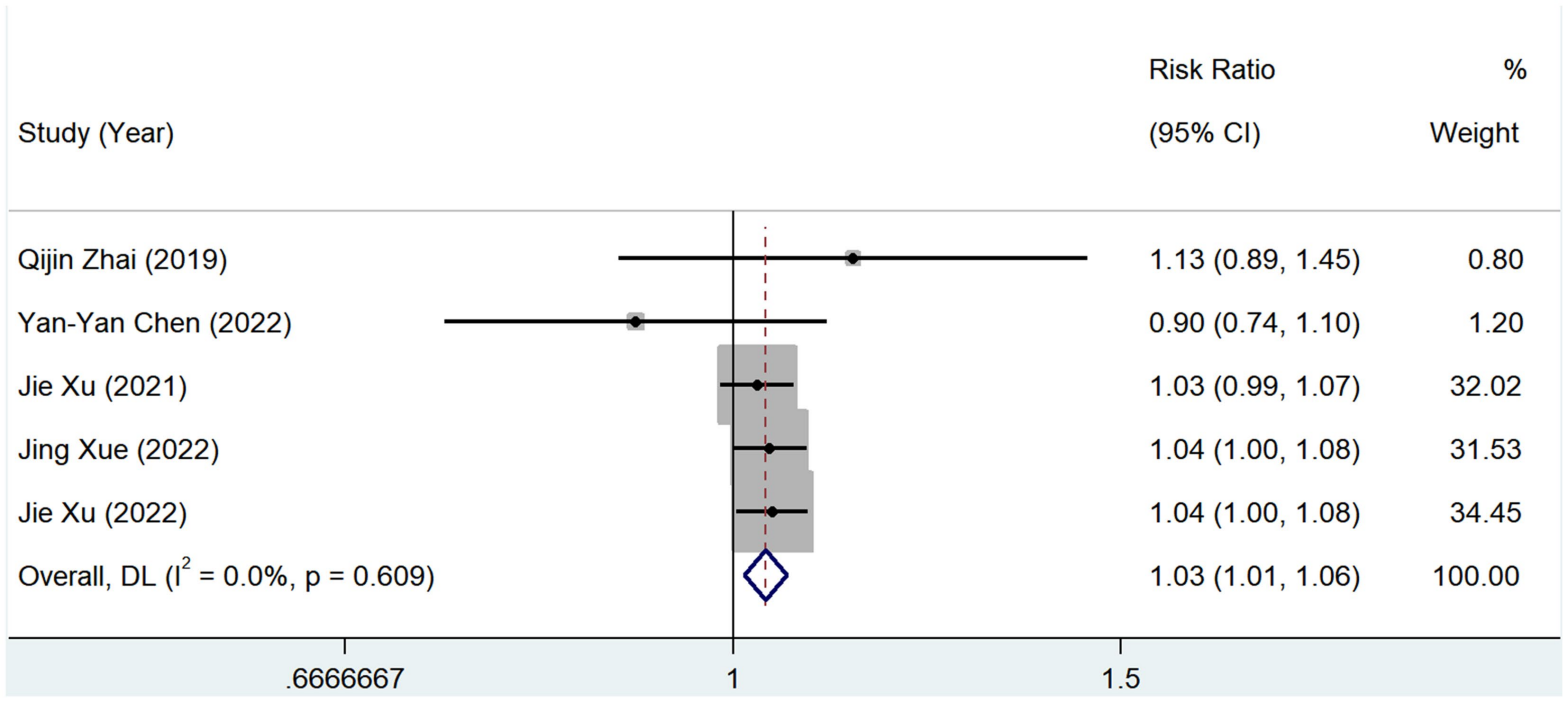
Relationship between TMAO and morbidity of hypertension.

In total, 5 studies reported the prevalence of hypertension between groups with different serum TMAO levels. The heterogeneity test showed that *p* = 0.609 and *I*^2^ = 0.0%, and the results of the fixed-effects model (RR, 1.035; 95% CI, 1.012–1.057; *Z* = 3.031; *p* = 0.002) showed that the prevalence of hypertension increased in patients with high levels of serum TMAO (Figure 7).

#### 3.4.2 Diabetes

**Figure 8:**
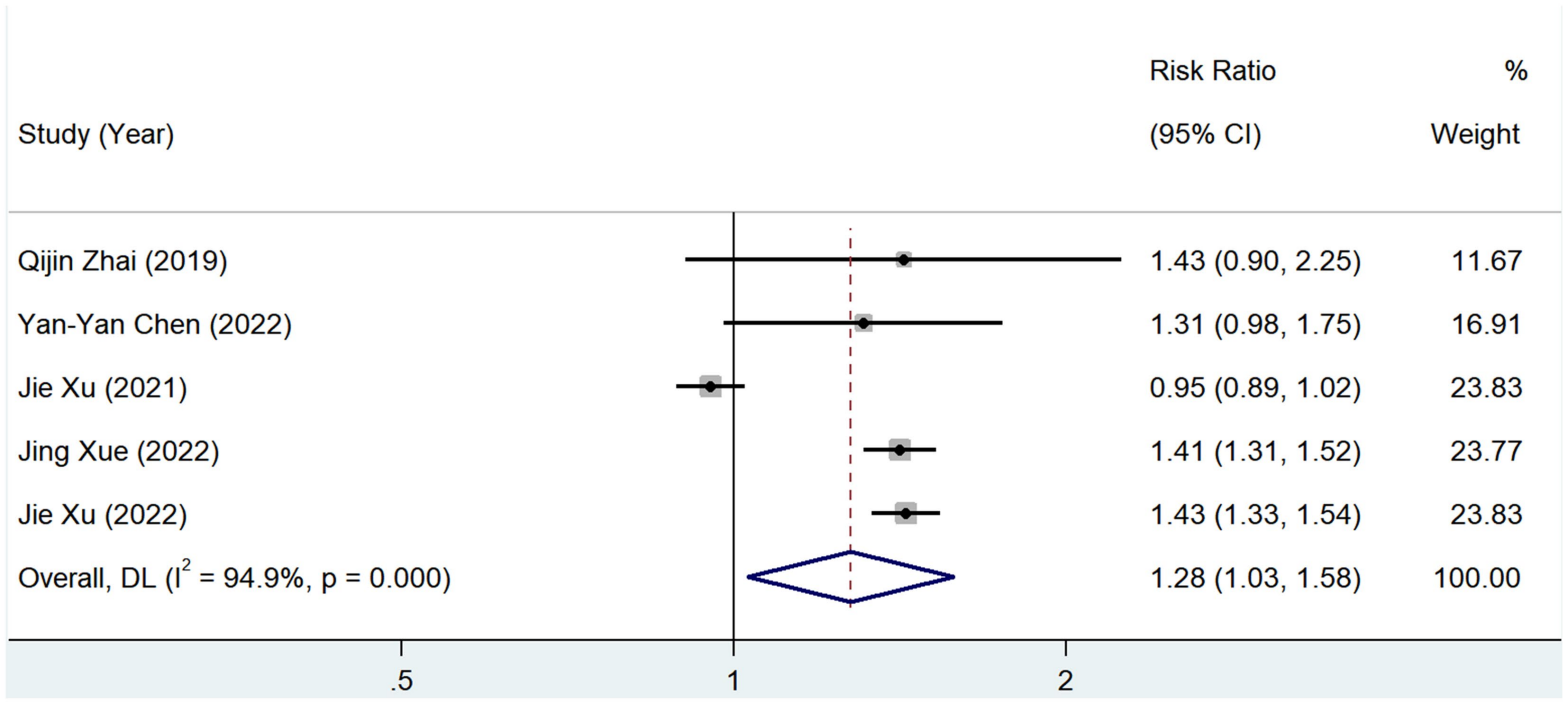
Relationship between TMAO and morbidity of diabetes.

In total, 5 studies reported the prevalence of diabetes among groups with different serum TMAO levels, and the test of heterogeneity showed *p* = 0.00, *I*^2^ = 94.9%. The results of a random-effects model [RR = 1.276, 95% CI (1.029–1.583), *Z* = 2.225, *p* = 0.026] showed that the prevalence of diabetes mellitus was elevated in patients with a high level of serum TMAO (Figure 8).

#### 3.4.3 Coronary heart disease

**Figure 9:**
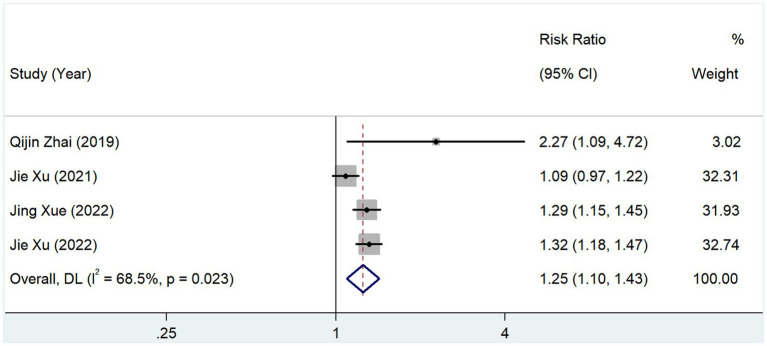
Relationship between TMAO and morbidity of coronary heart disease.

In total, 4 studies reported the incidence of coronary heart disease in groups with different serum TMAO levels. The test for heterogeneity showed that *p* = 0.023 and *I*^2^ = 68.5%, and the results of the random-effects model (RR, 1.249; 95% CI, 1.095–1.426; *Z* = 3.310; *p* = 0.001) suggested that the incidence of coronary heart disease was elevated in patients with high levels of serum TMAO (Figure 9).

#### 3.4.4 History of cerebral infarction

**Figure 10:**
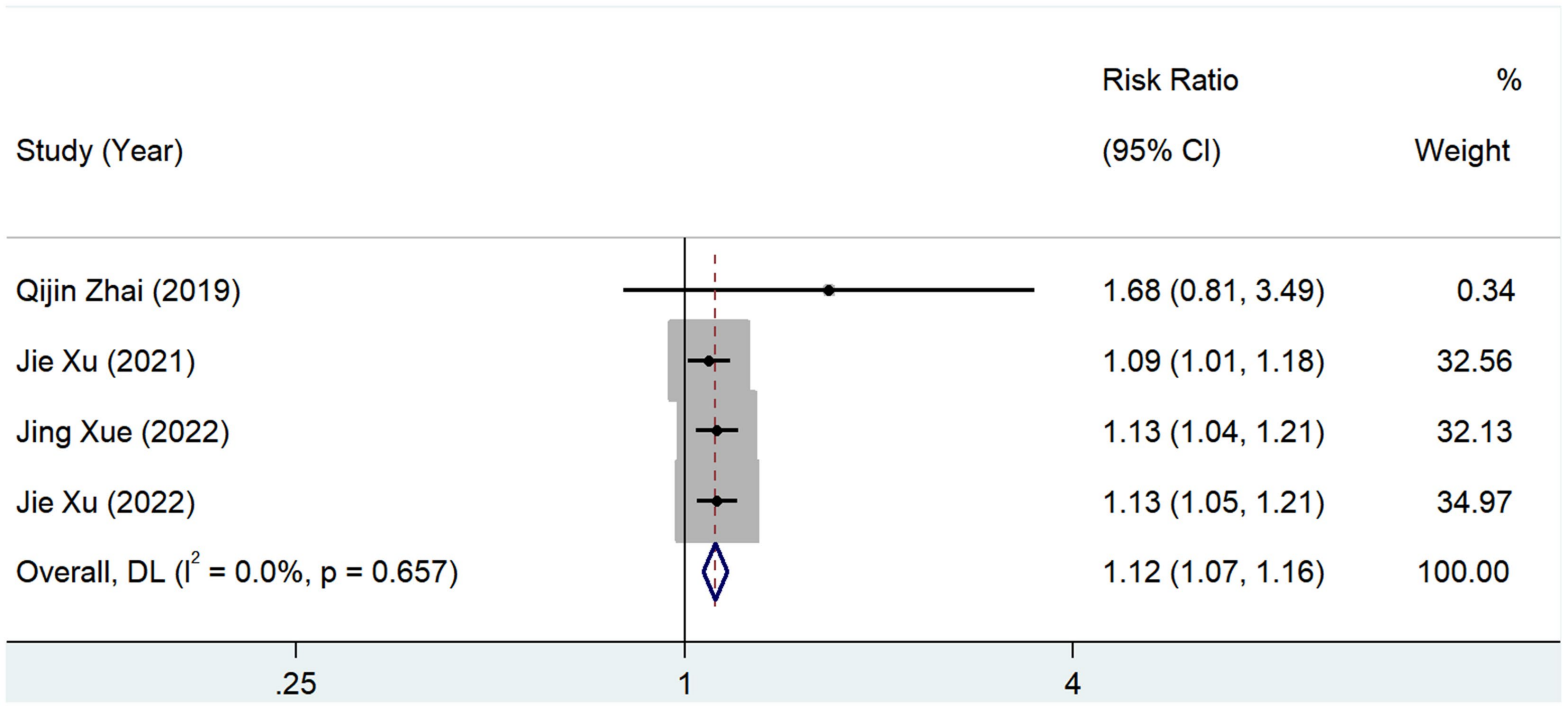
Relationship between TMAO and morbidity of cerebral infarction.

In total, 4 studies reported the prevalence of prior cerebral infarction in patients with different serum TMAO levels. The test of heterogeneity showed that *p* = 0.657 and *I*^2^ = 0.0%, and the results of the fixed-effects model (RR, 1.116; 95% CI, 1.069–1.165; *Z* = 5.044; *p* = 0.000) showed that the prevalence of cerebral infarction was high in patients with high levels of serum TMAO (Figure 10).

#### 3.4.5 Hyperlipidemia

**Figure 11:**
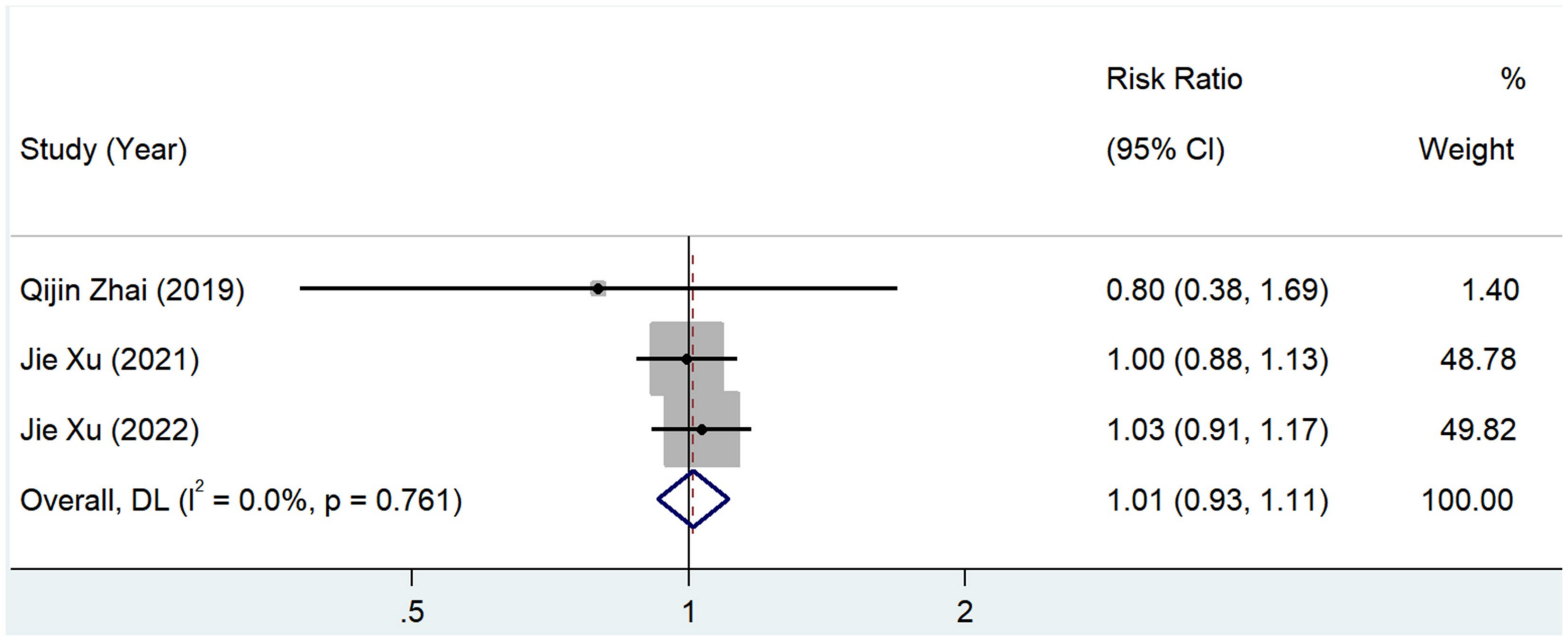
Relationship between TMAO and morbidity of hyperlipidemia.

In total, 3 studies reported the prevalence of hyperlipidemia between groups with different serum TMAO levels, and the test for heterogeneity showed that *p* = 0.761 and *I*^2^ = 0.0%. The results of the fixed-effects model (RR, 1.012; 95% CI, 0.926–1.106; *Z* = 0.256; *p* = 0.798) showed no significant correlation between TMAO and the prevalence of hyperlipidemia (Figure 11).

### 3.5 Basic features of risk prediction models

Of the 14 included studies, 4 reported six risk prediction models of TMAO for cerebral infarction, all of which used logistic regression analysis to construct risk prediction models, and the AUC of the six risk prediction models ranged from 0.66 to 0.8. Five risk prediction models had good predictive value (AUC ≥ 0.7). As shown in Table 3, the predictors were major cerebrovascular events at 90 days, mRS score of >2, mortality, and cerebral infarction incidence.

**Table 3:** The basic characteristics of the risk prediction model.

References	Modeling quantity	Modeling approach	AUC	95% confidence interval	Predictive factor
Tan et al. (10)	1	Logistic regression	0.72	0.61–0.83	Major cerebrovascular events in 90 days
Zhang et al. (11)	2	Logistic regression	0.78	0.72–0.83	mRS>2
			0.8	0.74–0.87	Mortality
Rexidamu et al. (13)	2	Logistic regression	0.66	0.62–0.71	Onset of cerebral infarction
			0.75	0.69–0.81	mRS>2
Chen et al. (19)	1	Logistic regression	0.78	0.75–0.82	Onset of cerebral infarction

**Item No.**	**Recommendation**	**Reported on Page No.**
**Reporting of background should include**		
1	Defined in the introduction	1
2	Stated in the introduction	1
3	Described in the introduction	1
4	Exposure or intervention is the level of the TMAO	1
5	Observational study	1
6	Described in the introduction	1
**Reporting of search strategy should include**		
7	Described in the Materials and Methods	2.3
8	Stated in the “Literature search”	2.1
9	Yes	2.1
10	Yes	2.1
11	Yes	2.1
12	Yes	2.1
13	Yes	2.1
14	Only English articles were searched	2.1
15	Handling abstracts and unpublished studies were excluded	2.2
16	Stated in the “data organization”	2.3
**Reporting of methods should include**		
17	Described in the methods	2
18	Stated in the methods	2
19	Stated in the “data extraction and quality evaluation”	2.3
20	Case–control studies and cohort studies were counted separately	2.5
21	Two researchers simultaneously applied the NOS scale to evaluate the quality of the literature, evaluated the quality of the literature	2.3
22	*I*^2^ is a parameter for quantitative analysis of the heterogeneity between the results of each study, which is distributed from 0 to 100%. When *I*^2^ > 50%, it indicates large heterogeneity	2.5
23	Described in the statistical methods. When *I*^2^ > 50%, it indicates large heterogeneity. When *p* > 0.1 and I2 ≤ 50%, the fixed effect model is applied. When *p* ≤ 0.1 and *I*^2^ > 50%, the random-effects model is applied	2.5
24	Yes	2
**Reporting of results should include**		
25	Yes	3.3
26	The descriptive information was performed in Table 1	3.1
27	We performed a subgroup analysis	3.4
28	We used a heterogeneity test	3
**Reporting of discussion should include**		
29	Discussed in the discussion	4.1
30	Discussed in the discussion	4
31	We evaluated the quality of the selected literature	4
**Reporting of conclusions should include**		
32	The findings may be influenced by underlying diseases	5
33	We have generalized the conclusions	5
34	We hope that more related studies can be carried out in the future to further explore its mechanism of action	5
35	Stated in the Acknowledgments	6

### 3.6 Funnel plot

No funnel plot analysis was performed because <10 articles were included in the literature for each analysis module.

## 4 Discussion

The role of TMAO in cerebrovascular diseases has been gradually receiving more attention. Current studies (25) have suggested that the dysregulation of the gut bacterial ecology is closely related to hypertension, diabetes, obesity, dyslipidemia, and metabolic syndrome. Some studies have also found that TMAO influences the occurrence, development, and prognosis of cerebral infarction to a certain degree (26), but the evidence is not yet conclusive. This review included 15 studies for which quality was guaranteed and the results were credible.

### 4.1 TMAO is a risk factor for the development of cerebral infarction and poor prognosis

The results of this study showed that TMAO is a risk factor for poor prognosis and morbidity due to cerebral infarction, which is consistent with the findings of a previous study (27). Furthermore, two studies (13, 24) have reported that TMAO is a risk factor for moderate-to-severe cerebral infarction, and an additional study (28) reported that TMAO is a risk factor for fluctuations in the acute phase of cerebral infarction (finding a 2-point increase in the NIHSS score over 3 days). In addition, four studies (12, 16, 20, 23) reported a positive correlation between TMAO and a history of cerebral infarction, which further validated the correlation between TMAO and cerebral infarction. All results showed that TMAO influenced the onset, severity, fluctuation, and prognosis of cerebral infarction.

Analysis of the logistic risk prediction models constructed in the included literature revealed that TMAO had a good predictive value for mRS scores of >2 at 90 days of onset (11, 13), which provides favorable evidence that TMAO is a risk factor for a poor prognosis of cerebral infarction. Rexidamu et al. (13) and Chen et al. (14) reported the predictive value of TMAO for the development of cerebral infarction, verifying that TMAO is a risk factor for the development of cerebral infarction, which aligns with the results of the present meta-analysis. Although the results of this meta-analysis showed no significant correlation between TMAO and mortality after cerebral infarction, Zhang et al. (12) confirmed that TMAO was a risk factor for increased mortality after cerebral infarction using the logistic risk prediction model. The inconsistency between these two results may be related to the small number of included studies and the small sample size. Tan et al. (10) found that TMAO is a risk factor for major cerebrovascular events (cerebral infarction, myocardial infarction, or death due to a cerebral infarction event) 90 days after cerebral infarction, further corroborating the negative impact of TMAO on the poor prognosis of cerebral infarction.

TMAO levels are influenced by diet, ethnicity, and genetics. In the Chinese population, plasma TMAO concentrations in healthy subjects are 2.33 μmol/L (17), compared with 4.4 μmol/L (26) in the United States, 5.6 μmol/L in the United Kingdom (29), 3.2 μmol/L in the Netherlands (30), and 20.4 μmol/L in Canada (31). European and American populations have much higher plasma TMAO levels than those of the Chinese populations. These differences may stem from variations in dietary structure between China, Europe, and the United States. Studies have reported that increased TMAO concentrations are associated with dietary intake of phosphatidylcholine and L-carnitine, which are more typically found in Western diet ingredients, such as red meat and full-fat dairy products (32). In addition to diet, the ethnic diversity of gut flora in the population may also contribute to this difference (33). The number of studies and sample size included in this meta-analysis were both small; these issues need to be verified in future clinical studies with a larger sample size.

The mechanisms underlying the role of TMAO in cerebrovascular diseases are poorly understood. Current research suggests the involvement of the following mechanisms. First, TMAO can promote vascular inflammation and oxidative stress (34, 35), leading to endothelial dysfunction and atherosclerosis (33), which damage cerebral blood vessels. Second, TMAO can mediate vascular inflammation through the sirtuin-3 (SIRT3)-superoxide dismutase (SOD)-mitochondrial reactive oxygen species (mtROS) signaling pathway to activate NLRP3 inflammatory vesicles mediating vascular inflammation (36). Third, TMAO can directly lead to high platelet hyperreactivity and enhanced risk of thrombosis (37).

### 4.2 TMAO is strongly associated with underlying disease

This study found that TMAO was significantly correlated with hypertension, diabetes mellitus, coronary artery disease, and cerebral infarction, but was not correlated with hyperlipidemia. Some studies have shown that TMAO has a stronger correlation with patients with hypertension or diabetes mellitus, both of which are risk factors for cerebral infarction (38, 39). The results of this study confirm these results.

A previous meta-analysis found that individuals with high TMAO levels had a significantly higher prevalence of hypertension than the normal population (39). Some studies suggest that intestinal flora can activate the immune system, intestinal sympathetic nervous system, and central nervous system through their metabolites, which interact with each other and affect the control of blood pressure (40). Animal experiments have shown that increased TMAO plasma levels in spontaneously hypertensive rats (SHRs) lead to increased plasma osmolality, which triggers the TMAO-AVP-AQP-2 axis to increase water reabsorption by the kidneys, thereby increasing circulating blood volume and elevating blood pressure (41).

TMAO plays an important role in the onset, development, and progression of diabetes and related complications. A previous meta-analysis showed a dose-dependent positive correlation between plasma TMAO concentration and the risk of developing diabetes mellitus (38). A prospective cohort study of older Chinese adults showed higher baseline serum TMAO concentrations in patients diagnosed with type 2 diabetes after a median follow-up of 8.9 years (42). A case–control study showed that TMAO metabolites were associated with the risk of developing type 2 diabetes, the composition of the gut flora was altered in patients with obesity and diabetes, compared with healthy subjects, and that dysbiosis of the gut flora could accelerate the development of diabetes (43). Animal studies have shown that TMAO impairs glucose tolerance by mediating insulin signaling pathways in the liver and upregulating proinflammatory mediators expressed in adipose tissue. The intestinal flora profile is complex and diverse, and it plays a crucial role in tissue and organ homeostasis and function, as well as the development of disease (44).

The results of the current study suggested no significant correlation between TMAO and hyperlipidemia; only three studies included in this meta-analysis involved the correlation between TMAO and hyperlipidemia. At present, relatively few clinical and basic studies investigate this issue. Recent studies have shown that TMAO can inhibit reverse cholesterol transport, leading to arterial cholesterol deposition and accelerated atherosclerotic lesions (45). However, the relationship between TMAO and lipid metabolism needs to be confirmed by large-scale clinical studies and further explored through more in-depth mechanistic studies.

We conducted a systematic evaluation and meta-analysis of the included studies using a standardized protocol and identified strict inclusion criteria to ensure that our analysis covered all available high-quality studies. Although the quality of the literature was high, the quantity was small. In each analysis, module <10 articles were included. The data were extracted from only two countries, which could have caused bias in the results. However, positive results were obtained throughout the meta-analysis, and the results were consistent and representative. In addition, TMAO is closely related to hypertension, coronary heart disease, diabetes mellitus, and other underlying diseases of cerebral infarction. These conditions may have influenced our results. In the future, as the literature on the relationship between TMAO and cerebral infarction continues to grow, a meta-analysis incorporating more literature and a larger sample size should be conducted.

## 5 Conclusion

TMAO is an independent risk factor that affects the onset, severity, progression, and prognosis of cerebral infarction. This study confirms the importance of TMAO and provides clues and ideas for TMAO-targeted cerebral infarction prevention and treatment. Further related studies should be carried out in the future to further explore the mechanism of action of the influence of TMAO on cerebral infarction.

## Data availability statement

The original contributions presented in the study are included in the article/supplementary material, further inquiries can be directed to the corresponding author.

## Author contributions

LW: Writing – original draft, Writing – review & editing. YN: Data curation, Writing – original draft, Writing – review & editing. WZ: Data curation, Software, Writing – original draft. SW: Conceptualization, Data curation, Formal analysis, Funding acquisition, Investigation, Project administration, Software, Supervision, Writing – original draft, Writing – review & editing.
